# Cost savings from primary care clinic continuity among patients with chronic disease in Alberta, Canada: a retrospective population-level cross-sectional study

**DOI:** 10.3399/BJGP.2026.0085

**Published:** 2026-09-22

**Authors:** Terrence McDonald, Brendan Cord Lethebe, Lee A Green

**Affiliations:** 1 Departments of Family Medicine and Community Health Sciences, Cumming School of Medicine, University of Calgary, Calgary, Alberta, Canada; 2 Clinical Research Unit, University of Calgary, Calgary, Alberta, Canada; 3 Department of Family Medicine, University of Alberta, Edmonton, Alberta, Canada; 4 Institute for Healthcare Policy and Innovation, University of Michigan, Ann Abor, Michigan, USA

**Keywords:** continuity of patient care, cost analysis, general practice, health service utilisation., health care costs

## Abstract

**Background:**

Continuity of care with a primary care physician (PCP) is well known to improve patient health, result in less health service utilisation (HSU), and lower costs. Less is known about the impact of clinic continuity related to team-based care and shared practice models. Information on the cost impact of such models is needed to guide design and payment policies.

**Aim:**

To estimate savings in HSU costs for patients with chronic disease when they are moved from primary care clinics with low and moderate continuity levels to those with high levels.

**Design and setting:**

A 3-year retrospective population based cross-sectional study examined the HSU costs associated with three levels of primary care clinic continuity for approximately 70 000 matched patients from a cohort of 500 000 patients with chronic disease in Alberta, Canada. All fee-for-service PCPs were included.

**Method:**

Clinic continuity was calculated by adapting the Usual Provider of Care (UPC) Index. Costs were estimated using costing methodologies from the Canadian Institute of Health Information.

**Results:**

The association between continuity and acute care costs suggests achieving high clinic continuity (UPC 80–100%) for all 195 000 patients in less than high clinic continuity care could theoretically yield upwards of £898 million over 3 years. Estimated per-patient gains were highest among the most medically complex patients.

**Conclusion:**

Cost savings were associated with clinic continuity among patients with common chronic diseases at all levels of complexity. Significant cost savings could be realised by focusing patient care on primary care clinics that favour high levels of continuity.

## How this fits in

There is great emphasis being placed on developing team-based primary care and shared care models that incentivise both patient access and continuity of care. This study provides valuable cost information on the potential saving when community primary care clinics (versus individual primary care physicians) provide high levels of continuity of care.

## Introduction

Continuity of care with an individual primary care physician (PCP) is associated with lower costs and better patient health outcomes.^1–4^ PCP continuity is known to reduce patient mortality, emergency department visits, hospital stays, and the overuse of procedures as well as improve patient satisfaction.^5–8^ Less is known about primary care clinic-level continuity and its impact on health service utilisation (HSU) and associated health system costs for patients with chronic disease.^9^

Within the current context of Canada’s PCP shortage and inverse care law for many patients in need of accessible care,^10,11^ health funders are placing much emphasis on the attachment of patients to a PCP.^12^ However, the trend towards part-time practice, reduced levels of provider continuity, and competing virtual care platforms work against these initiatives.^13,14^ To counter this, there is growing interest in models of care that incentivise both access and continuity of care with a primary care team.^15^ Notably, the investment in and uptake of such team-based models call for cost information on their potential impact. The aim of this study was to offer new information and insight into the potential cost savings when clinics (versus individual providers) provide high levels of continuity of care.

Building on the authors’ previous work, which explored the contribution of both provider and clinic continuity,^6^ the current study focused its attention on the question of comparing the costs of HSU among varying levels of primary care clinic continuity. The findings are timely and will inform current policy discussions within Canadian jurisdictions,^16^ and offer some new insight into understanding of this relationship between clinic continuity, patient care outcomes, as well as HSU and costs.

## Method

### Design, study population, and setting

A population-based retrospective cohort design was used to examine the association between primary care clinic continuity and HSU. This prevalence-based analysis included Alberta patients with International Classification of Disease ICD-9 and ICD-10 codes for heart failure, chronic obstructive pulmonary disease (COPD), ischaemic heart disease (IHD), diabetes mellitus, and asthma between 1 April 2015 and 31 March 2018. Each disease (case) definition used validated algorithms and required two physician visits with the diagnostic code(s) within a 2-year period or one hospital discharge with the disease code(s) in any diagnosis field or intervention code in any diagnostic field (See Supplementary Table S1).^17,18^ Patients with ≥3 PCP visits who also met the diagnostic disease criteria for at least one of the five conditions prior to the beginning of the 2017–2018 fiscal year were included.

Primary care clinic continuity was calculated by applying the Usual Provider of Care (UPC) Index calculation to clinic level rather than individual physician. In this case, the proportion of visits to the clinic (patient’s usual PCP at main clinic + patient’s usual PCP at any facility other than main clinic + all other PCPs at same main clinic) serves as the numerator and all visits to any PCP serves as the denominator.^19,20^ PCPs were assigned a single clinic as their ‘main‘ clinic based on their highest frequency of claims.

### Perspective

An Alberta health system perspective was used to analyse the impact on overall HSU including costs for inpatient stays, specialist physician services, and PCP visits. In 2017/18, there were approximately 4038 PCPs practicing in Alberta. More than 80% of PCPs in Alberta are remunerated by a fee-for-service payment model, the highest in Canada.^21^ PCPs serve as gatekeepers to the greater health system that includes seeking medical and surgical consultations with specialty providers for their patients. Patients in Alberta are free to see any PCP, in any practice, and are not constrained by a registry. Similar to other provinces and territories in Canada, the Alberta Ministry of Health pays for all medically required services provided by a physician as part of a single payer system through the Alberta Health Care Insurance Plan.^22^

### Data sources

Unique lifetime (patient) identifiers were linked to the Primary and Preventative Health Services administrative databases to estimate utilisation of:

- inpatient services (hospital stays) from the discharge abstract database;
- emergency department visits from the National Ambulatory Care Reporting System; and
- physician services (PCP and specialist visits) from the Alberta Health Care Insurance Plan practitioner claims database.^23–25^

The linking identifiers were coded such that patients could not be identified.

### Propensity matching

To reduce potential bias because of clinical and demographic characteristics, propensity score matching was conducted, and results were stratified by clinical risk grouper (CRG). CRG is a well-established claims-based methodology that uses historical clinical and demographic information to classify patients into specific risk categories. It includes a numerical ranking,^1–9,26^ from least CRG 1 to most risk CRG 9, and can be used as a predictor of future HSU. In this study, patients were categorized into one of three CRG groupings to assign similar complexity levels: high CRG (5-9), moderate (3-4) and low (1-2).^27^

Specifically, matching was done with 1:1:1 ratio on the clinic UPC Index category variable with three categories (0–39, 40–79, 80–100) using nearest neighbour matching, with a tight caliper (<0.000000001) for exact matching. Variables used in propensity score calculations included: patient gender, patient age, geographic area using a seven-category rural–urban continuum,^28^ Charlson Comorbidity Index, and binary indicators of disease states for heart failure, COPD, IHD, asthma and diabetes. Balance was assessed by comparing Rubin’s B and R in the unmatched and matched cohorts.^29,30^

### Cost calculations

Costs were estimated for the matched cohort using both the Canadian Institute of Health Information Case Mix Grouping Plus and Comprehensive Ambulatory Classification System methodologies for inpatient and emergency department visits.^31,32^ Specifically, these standardised methods assign a cost to each hospital episode or emergency department visit equal to the resource intensity weight multiplied by the cost per standard hospital stay.^33^ The costs for PCP services were the actual paid amounts for claims made to the Alberta Health Care Insurance plan, which includes time of nurses and other staff who were working for the physicians.^25^ These costs were calculated individually for each of the 2015–16, 2016–17, and 2017–18 fiscal year and combined.

### Outcomes

The study outcome was the mean total per-patient cost of emergency department visits, hospital stays, and PCP and specialist visits for patients with COPD, heart failure, IHD, asthma, and diabetes. Cost differences in each health service as well as total cost were estimated comparing matched mean costs for high continuity versus low and medium clinic continuity. These results were stratified by CRG levels. Estimated potential cost savings to Alberta of moving all low and medium continuity patients to high continuity were estimated by multiplying the total number of patients in each group (continuity by CRG level) by the cost difference to that respective group.

### Statistical analysis

Descriptive univariable analyses were used to compare patients’ demographic, HSU, and associated cost outcomes by level of clinic continuity. Percentages were calculated for categorical variables, and means were calculated for numeric variables. Matched mean cost differences were calculated between low and moderate levels of clinic continuity (UPC 0–39 and UPC 40–79) relative to high clinic continuity (UPC 80–100), within CRG categories.

### Sensitivity analysis

Given cost data are typically skewed owing to high-cost outlier cases, a sensitivity analysis was conducted that used a non-parametric approach, calculating means and confidence intervals (CIs) using a bootstrap procedure with 1000 iterations, stratified by both CRG (1–2, 3–4, and 5–9) and clinic continuity categories.^34,35^

The bootstrapping procedure was repeated to calculate mean cost differences between low and moderate levels of clinic continuity (0–39 and 40–79) relative to high clinic continuity (80–100), within CRG categories. All 2018 Canadian dollars were converted to British Pounds using the 2018 exchange rate^36^ (in addition for some calculations 2018 Canadian dollars were also converted to US dollars using the 2018 exchange rate^37^). The analysis was conducted using R version 4.4.2 (https://cran.r-project.org/).

## Results

A total of 68 625 exactly matched patients were identified from a total cohort of 494 902 patients with chronic disease in Alberta between 2015 and 2018 (Table 1). These matched patients were equally distributed across continuity levels for all variables owing to exact matching. A greater proportion of patients were female than male (55.2% [*n* = 37 875/68 625] and 44.8% [*n* = 30 750/68 625], respectively). Overall, most patients were between the ages of 15 and 44 (47.5% [*n* = 32 586/68 625]) years, followed by ages 45–64 (31.1% [*n* = 21 366/68 625]), 65–74 (13.4% [*n* = 9162/68 625]), and ≥75 years (8.0% [*n* = 5511/68 625]). Asthma was the most common of the chronic conditions (55.8% [*n* = 38 292/68 625]), followed by diabetes (27.2% [*n* = 18 699/68 625]), IHD (19.1% [*n* = 13 131/68 625]), COPD (8.9% [*n* = 6135/68 625]), and heart failure (8.6% [*n* = 5895/68 625]). The mean Charlson Comorbidity Index was 0.28 (standard deviation [SD] 0.69). Most of the medically complex patients, with 45.4% (*n* = 31 143/68 625) in CRG 5–9 (Table 2).

The unmatched population was not equally distributed across continuity levels. There were 195 000 patients in low and medium continuity, with 66 214/494 902 (13.4%) had 0–39% clinic continuity, 128 741/494 902 (26.0%) had 40–79% clinic continuity, and 299 947/494 902 (60.6%) had 80–100% clinic continuity. Of note, the mean Charlson Comorbidity Index for unmatched patients was higher at 0.37 (SD 0.82) and there was a higher proportion in CRG 5–9 (53.4% [*n* = 264 157/494 902]) and a lower proportion with low complexity CRG 1–2 (33.8% [*n* = 167 114/494 902]) (see Supplementary Table S2). In addition, relative to the matched cohort, there were more patients with diabetes (40.9% [*n* = 202 222/494 902]), IHD (28.0% [*n* = 138 519/494 902]), heart failure (10.8% [*n* = 53 414/494 902]), and COPD (13.2% [*n* = 65 434/494 902]), and fewer with asthma (37.7% [*n* = 186 368/494 902]) (Supplementary Table S2).

**Table 1.** Matched patient demographics

Variable	Overall (*N* = 68 625)	UPC 0–39%(*n* = 22 875)	UPC 40–79% (*n* = 22 875)	UPC 80–100% (*n* = 22 875)	SMD
**Sex, male**	30 750 (44.8)	10 250 (44.8)	10 250 (44.8)	10 250 (44.8)	<0.001
**Age, years, mean (SD)**	52.0 (22.1)	52.0 (22.1)	52.0 (22.1)	52.0 (22.1)	<0.001
**Age group, years**					<0.001
15–44	32 586 (47.5)	10 862 (47.5)	10 862 (47.5)	10 862 (47.5)	
45–64	21 366 (31.1)	7122 (31.1)	7122 (31.1)	7122 (31.1)	
65–74	9162 (13.4)	3054 (13.4)	3054 (13.4)	3054 (13.4)	
≥75	5511 (8.0)	1837 (8.0)	1837 (8.0)	1837 (8.0)	
**Geography**					<0.001
Metro	42 003 (61.2)	14 001 (61.2)	14 001 (61.2)	14 001 (61.2)	
Moderate metro influence	8970 (13.1)	2990 (13.1)	2990 (13.1)	2990 (13.1)	
Moderate urban influence	1233 (1.8)	411 (1.8)	411 (1.8)	411 (1.8)	
Rural	7827 (11.4)	2609 (11.4)	2609 (11.4)	2609 (11.4)	
Rural centre	1755 (2.6)	585 (2.6)	585 (2.6)	585 (2.6)	
Rural remote	1182 (1.7)	394 (1.7)	394 (1.7)	394 (1.7)	
Urban	5655 (8.2)	1885 (8.2)	1885 (8.2)	1885 (8.2)	
**Comorbidities**					
CCI mean (SD)	0.28 (0.69)	0.28 (0.69)	0.28 (0.69)	0.28 (0.69)	<0.001
Diabetes	18 699 (27.2)	6233 (27.2)	6233 (27.2)	6233 (27.2)	<0.001
Heart failure	5895 (8.6)	1965 (8.6)	1965 (8.6)	1965 (8.6)	<0.001
COPD	6135 (8.9)	2045 (8.9)	2045 (8.9)	2045 (8.9)	<0.001
Asthma	38 292 (55.8)	12 764 (55.8)	12 764 (55.8)	12 764 (55.8)	<0.001
IHD	13 131 (19.1)	4377 (19.1)	4377 (19.1)	4377 (19.1)	<0.001

Data are* n* (%) unless otherwise specified. CCI = Charlson Comorbidity Index. COPD = chronic obstructive pulmonary disease. IHD = ischaemic heart disease. SD = standard deviation. SMD = standardised mean difference.

**Table 2.** Matched number of patients with chronic disease by complexity and facility continuity^a^

UPC	**Clinic Continuity UPC Index**			
	0–39	40–79	**≥**80	Total
**Matched cohort**				
1–2	9767 (42.7)	9767 (42.7)	9767 (42.7)	29 301 (42.7)
3–4	2727 (11.9)	2727 (11.9)	2727 (11.9)	8181 (11.9)
5–9	10 381 (45.4)	10 381 (45.4)	10 381 (45.4)	31 143 (45.4)
Total	22 875 (33.3)	22 875 (33.3)	22 875 (33.3)	68 625 (100)
**Unmatched cohort**				
1–2	21 281 (12.7)	47 563 (28.5)	98 270 (58.8)	167 114 (33.8)
3–4	7197 (11.3)	17 668 (27.8)	38 766 (60.9)	63 631 (12.9)
5–9	37 736 (14.3)	63 510 (24.0)	162 911 (61.7)	264 157 (53.4)
Total	66 214 (13.4)	128 741 (26.0)	299 947 (60.6)	494 902 (100)

Data are *n* (%). CRG = clinical risk grouper. UPC = Usual Provider of Care.

^a ^ Note that the % data sum by row and column, respectively.

Logistic regression coefficients for propensity score matching can be found in Supplementary Tables S3 and S4. The balance test shows that bias was reduced by exact matching (Supplementary Figures S1 and S2). Specifically, the mean bias was reduced to zero in both matches.

The mean HSU costs per matched patient over the 3-year period are shown in Table 3. Hospital stays accounted for 45–84% of total costs. Emergency department, PCP, and specialist visits costs were less than the hospital stay costs and followed the same gradient as hospital stay costs across CRG and clinic continuity.

**Table 3.** Three-year averagemean HSU and costs by CRG and facility continuity

Events and costs	Patient complexity CRG 1–2			Patient complexity CRG 3-4			Patient complexity CRG 5–9		
	**Clinic UPC Index,** 0–39	**Clinic UPC Index, 40–79**	**Clinic UPC Index, ≥80**	**Clinic UPC Index,** 0–39	**Clinic UPC Index,** 40–79	**Clinic UPC Index, ≥80**	**Clinic UPC Index,** 0–39	**Clinic UPC Index,** 40–79	**Clinic UPC Index, ≥80**
**HSU, events per person per year, *n* (95% CI)**									
ED visits	0.86 (0.83 to 0.89)	0.77 (0.75 to 0.80)	0.58 (0.56 to 0.60)	1.48 (1.41 to 1.56)	1.24 (1.18 to 1.31)	1.03 (0.97 to 1.10)	2.36 (2.29 to 2.44)	1.95 (1.90 to 2.01)	1.47 (1.43 to 1.52)
Hospital stays	0.09 (0.09 to 0.10)	0.07 (0.07 to 0.08)	0.06 (0.06 to 0.06)	0.15 (0.14 to 0.16)	0.11 (0.10 to 0.12)	0.10 (0.09 to 0.11)	0.54 (0.53 to 0.56)	0.40 (0.39 to 0.41)	0.31 (0.30 to 0.32)
PCP visits	3.34 (3.28 to 3.39)	3.68 (3.63 to 3.74)	3.60 (3.53 to 3.66)	5.96 (5.76 to 6.17)	6.72 (6.53 to 6.91)	6.65 (6.47 to 6.84)	9.67 (9.44 to 9.90)	10.36 (10.14 to 10.57)	9.63 (9.44 to 9.82)
**Costs,^a^ £ (95% CI)**									
Hospital stays costs	1824.80 (1627.12 to 2022.44)	1081.91 (888.20 to 1275.63)	832.05 (742.29 to 921.81)	3232.25 (2744.49 to 3719.93)	1775.90 (1517.28 to 2034.53)	1909.49 (1095.70 to 2723.25)	21714.38 (20843.37 to 22601.39)	11587.48 (11060.71 to 12114.20)	7926.99 (7549.33 to 8304.80)
ED costs	719.95 (697.99 to 742.09)	673.38 (650.71 to 696.05)	543.32 (522.94 to 563.79)	1356.64 (1281.57 to 1431.79)	1208.18 (1146.17 to £1272.69)	1074.62 (1014.66 to 1134.89)	2432.77 (2366.17 to 2499.78)	2096.71 (2041.42 to 2149.99)	1722.79 (1674.27 to 1773.90)
PCP visit costs	502.56 (491.03 to 513.94)	515.78 (505.72 to 525.77)	455.11 (447.00 to 463.21)	883.97 (849.99 to 916.45)	933.50 (899.74 to 968.46)	860.69 (835.79 to 887.59)	1609.24 (1570.27 to 1647.64)	1434.94 (1406.12 to 1463.71)	1234.42 (1209.49 to 1259.24)
Specialist visit costs	46.10 (43.70 to 48.51)	42.08 (39.99 to 44.18)	33.96 (32.17 to 35.75)	91.53 (84.37 to 98.67)	83.68 (76.18 to 91.14)	62.37 (56.84 to 67.94)	230.67 (219.46 to 241.99)	174.49 (167.38 to 181.61)	126.60 (121.53 to 131.65)
Total cost	3092.29 (2884.75 to 3300.31)	2313.07 (2111.53 to 2512.20)	1865.93 (1764.52 to 1967.34)	5564.59 (5036.09 to 6091.19)	4000.06 (3712.11 to 4293.03)	3910.58 (3085.91 to 4724.87)	25 998.34 (25 073.68 to 26 938.80)	15 299.53 (14 729.33 to 15 848.51)	11 014.38 (10 596.84 to 11 423.91)

^a^Converted from Canadian to GBP using 2018 mean exchange rate of 1 CAD ≈ 0.57 GBP. CAD to GBP Exchange Rate History for 2018.^36^ CI = confidence interval. CRG = clinical risk grouper. ED = emergency department. HSU = health service utilisation. PCP = Primary Care Physician. UPC = Usual Provider of Care.

Differences in matched per-patient HSU costs between high continuity and low as well as medium continuity, across CRG levels, are shown in Table 4 (see Supplementary Table S5 for USD). The biggest difference in total HSU cost was in the CRG 5–9 group with low as compared with high continuity differing by £14 984 (95% confidence interval [CI] = £13 976 to 15 991)*.* The next biggest difference in total HSU cost was in the CRG 5–9 group with medium as compared with high continuity at £4283 (95% CI = £3585 to 4981), followed by CRG 3–4 with medium compared with high continuity at £1655 (95% CI = £677 to 2633). Differences in total HSU cost between low and high and medium and high clinic continuity for the CRG 1–2 group ranged from £1227 (95% CI = £996 to 1459) to £447 (95% CI = £222 to 672). The main driver of cost differences was hospital stay costs across all CRG groups, which were lower for the high continuity group compared with the medium or low continuity groups.

**Table 4.** HSU cost differences by clinic continuity and patient complexity

Cost category^a^ £ (95% CI)	Patient complexity CRG 1–2		Patient complexity CRG 3–4		Patient complexity CRG 5–9	
	**Clinic UPC Index, 0–39** versus 80–100	**Clinic UPC Index,** 40–79 versus 80–100	**Clinic UPC Index,** 0–39 versus 80–100	**Clinic UPC Index,** 40–79 versus 80–100	**Clinic UPC Index,** 0–39 versus 80–100	**Clinic UPC Index,** 40–79 versus 80–100
Hospital stay, £	991.65 (774.12 to 1209.17)	248.87 (34.84 to 462.89)	1321.79 (372.00 to 2271.58)	–135.77 (–990.54 to 719.01)	13 796.19 (12 837.76 to 14 754.62)	3663.29 (3012.64 to 4313.93)
ED, £	176.73 (146.02 to 207.44)	130.17 (99.82 to 160.52)	281.91 (185.10 to 378.72)	134.44 (46.66 to 222.23)	709.08 (625.95 to 792.22)	372.01 (298.73 to 445.30)
PCP, £	46.84 (32.98 to 60.70)	59.98 (46.67 to 73.29)	22.00 (–19.63 to 63.61)	72.51 (29.33 to 115.69)	374.20 (327.73 to 420.67)	199.96 (161.21 to 238.70)
Specialist, £	12.13 (9.10 to 15.16)	8.12 (5.37 to 10.88)	29.13 (20.08 to 38.18)	21.31 (11.99 to 30.64)	104.10 (91.81 to 116.39)	47.89 (39.16 to 56.63)
Total cost, £	1227.35 (995.90 to 1458.80)	447.14 (222.23 to 672.05)	1654.82 (676.83 to 2632.81)	92.50 (–780.71 to 965.70)	14 983.57 (13 975.73 to 15 991.40)	4283.15 (3585.00 to 4981.29)

a Converted from Canadian to GBP using 2018 mean exchange rate of 1 CAD ≈ 0.57 GBP. CAD to GBP Exchange Rate History for 2018.^36^ CI = confidence interval. CRG = clinical risk grouper. ED = emergency department. HSU = health service utilisation. UPC = Usual Provider of Care.

Based on the above estimated matched cost differences, the estimated potential cost savings to the province if all of these patients received high clinic continuity was £898 351 296 over a 3-year period (or £299 450 432 per year) (Table 5). The potential savings listed in this research amount to 13% of the total costs of acute care services for the province of Alberta.^38^

**Table 5.** Three-year potential cost savings if all patients received 80% to 100% clinic continuity

**Clinic continuity** and patient complexity	Patients, *n*	Average cost difference		Cost savings			
		£^a^	$USD^b^	£^a^	£ (SD)	$USD^b^	$USD (SD)
**Clinic UPC Index, 0–39%**							
CRG 1–2	21 281	1227 (996,1459)	1633 (1327,1944)	26 111 787 (21 195 876 to 31 048 979)	237 495 960	34 759 109 (28 239 887 – 41 370 264)	316 256 941
CRG 3–4	7197	1654 (677, 2633)	2204 (901,3507)	11 903 838 (4 872 369 – 18 949 701)	186 243 966	15 859 093 (6 484 497 – 25 239 879)	248 001 423
CRG 5–9	37 736	14984 (13976,15991)	19 955 (18618,21292)	565 436 224 (527 398 336 – 603 436 376)	1 842 611 144	753 012 823 (702 568 848 – 803 474 912)	2 453 632 456
**Clinic UPC Index, 40–79%**							
CRG 1–2	47 563	447 (222,672)	595 (296,894)	21 260 661 (10 558 986 – 31 962 336)	525 428 461	28 305 217 (14 078 648 – 42 521 322)	699 699 293
CRG 3–4	17 668	92 (-781,966)	123 (–1040,1287)	1 625 456 (–13 798 708–17 067 288)	407 812 776	2 175 991 (–18 374 720 – 22 738 716	543 061 316
CRG 5–9	63 510	4283 (3585,4981)	5703 (4776,6640)	272 013 330 (227 683 350 – 316 343 310)	2 106 626 700	362 226 745 (303 323 760 – 421 706 400)	2 805 236 700
**Total**	–	–	–	898 351 296	–	1 196 338 978	–

^a^Converted from Canadian to GBP using 2018 mean exchange rate of 1 CAD ≈ 0.57 GBP.^36^ ^b^Converted from Canadian to USD using mean 2018 exchange rate of 1 CAD ≈ 0.771 USD.^37^ CRG = clinical risk grouper. SD = standard deviation. UPC = Usual Provider of Care. Total = cost savings for clinic continuity 0%–39% + cost savings for clinic continuity 40%–79%.

To analyse the sensitivity of these results, unmatched per-patient costs as well as bootstrapped costs were also estimated. Matching was undertaken to estimate the costs differences between the three categories of clinic continuity (low 0–39%, medium 40–79%, and high 80–100%). The differences in cost per patient between matched and unmatched groups were similar meaning the estimated potential system cost savings would be similar as well (Supplementary Tables S6–S8 and Supplementary Figures S1 and S2). The differences in cost per patient between bootstrapped matched costs were small (Supplementary Table S9).

## Discussion

### Summary

This 3-year observational study of a matched cohort of approximately 70 000 patients with one of five chronic diseases and their PCPs demonstrated a positive association between receiving high continuity of care within a clinic (UPC Index ≥80%) and lower health system costs compared with low (UPC Index 0–39%) and medium (UPC Index 40–79) levels of clinic continuity. Within this observed association, the estimated potential gains per patient were highest when the most medically complex patients were moved from low and medium levels of clinical continuity. Significant gains per patient were also observed in less medically complex patients; the reason for this remains unclear and will benefit from more detailed exploration. The estimated results were different for the matched and unmatched cohorts, suggesting selection or temporal bias in the matched versus unmatched populations. Matching resulted in more conservative cost difference estimates, which may be attributed to differences across the two groups in terms of proportion of comorbidities and chronic disease and age.

### Strength and limitations

Several limitations as well as caveats in implications for policy and future research should be noted in this this study. Among limitations, first and foremost, this observational study cannot prove causation. Therefore, it will be important to conduct rigorous programme evaluation of policies and models that aim to increase clinic continuity as they are developed and scaled up. Given these policies and programmes have yet to be developed but are evolving in some jurisdictions, it was not possible to include implementation costs in this paper and that would be an important part of future work. Second in importance is that to estimate the potential cost savings to Alberta of high continuity care for patients with chronic disease in the current study it is assumed all patients can be provided with high continuity clinic care. This may not be feasible for several reasons, although the extent to which it is possible is difficult to assess without a concrete policy being evaluated, which could be the focus of future work. Third, other outpatient costs aside from emergency department visits and specialty visits (such as laboratory and imaging) were not included. However, these costs typically are a fraction of inpatient costs and would not have a meaningful impact on conclusions.^39^

First among caveats for policy and future work, the current analysis did not adjust for inflation to reflect current day cost values, therefore, the cost estimates might be undervalued to a small extent. Second, the study looked at only five of the most common chronic conditions, so the overall cost impact of continuity is likely greater than the estimates in the current study. Third, there may be differences in the level of continuity owing to differences in capacity and efficiency and the resulting ability to provide continuous care. The definition of continuity applied in the current study centred around PCPs providing care from one main clinic site along with patients seeking care from their main provider at multiple sites. Future work could explore the extent to which this occurs.

### Comparison with existing literature

There are numerous studies that have identified the association between lower levels of provider- and practice-level continuity and higher HSU and costs.^1,4^ Many of these studies were from a variety of nations and health system designs that applied a different measure or combination of continuity indices and outcomes to a variety of patient and provider cohorts, thereby limiting their generalisability.^40,41^ Other studies have highlighted a similar observational relationship between higher interpersonal continuity and patient attachment with less HSU and costs.^42–44^ However, there are few studies, if any, that explore the association of primary care clinic continuity and total costs at a health system level. To the authors’ knowledge, this study is the first to explore the association of primary care clinic continuity and health system costs at a population level. This study uniquely used an established national costing methodology with high-quality patient level (matched data) for an entire cohort of PCPs.

### Implications for research and practice

Substantial structural, policy, and payment changes within primary care and significant optimisation of existing resources would be needed to move more patients with chronic disease to ≥80% clinic continuity. Funding for primary care has historically amounted to 5% of total health spending in Canada, yet continuity in primary care has consistently been shown to result in better health for patients while reducing costs to the greater health system.^45^ Investing in models, such as the Patient’s Medical Home, that support team-based activities in keeping with the 4Cs of primary care (that is, first contact, comprehensiveness, coordination, and continuity) proposed by Starfield offers a path towards realising the potential savings.^46,47^ Focusing attention on enabling clinic members to work at the full scope of their skill set may do so as well. Both changes could improve access and move more patients to high clinic continuity, and moving more patients to a continuous care provider clinic team would have the added benefit of reducing strains on the acute care system. Furthermore, the achievement of such a care delivery model would ultimately result in less patient suffering. Several models now under development in several Canadian jurisdictions show potential towards this end.^48–54^Canada can learn from the experiences of other countries in this regard.^55^ Future research work that explores the constructs and nuances of high continuity providers and practices will be of great value.

In conclusion, lower costs were associated with higher clinic continuity among patients with five common chronic diseases at all levels of complexity in Alberta, Canada. Although the potential cost savings to the health system are hypothetical, they may be realised by enabling continuity of care with PCPs and group care practices that favour this model.

## Data

The data that formed the basis for this study was made available to the authors on a temporary basis by Primary and Preventative Health Services, and the authors no longer have access to it. Anyone wishing to view this data should make an access request to Primary and Preventative Health Services.
